# Understanding risk factors and prognosis in diabetic foot ulcers

**DOI:** 10.1515/biol-2025-1137

**Published:** 2025-08-08

**Authors:** Jixue Wang, Xirui Yang, Tao Zhou, Haitao Ma, Xingxing Yuan, Shuxun Yan

**Affiliations:** Department of Peripheral Vascular Medicine, First Affiliated Hospital of Henan University of Traditional Chinese Medicine, Zhengzhou, China; Department of Ophthalmology, The First Affiliated Hospital of Henan University of Traditional Chinese Medicine, Zhengzhou, China; Department of Gastroenterology, Heilongjiang Academy of Traditional Chinese Medicine, Harbin, China; Department of Endocrinology, First Affiliated Hospital of Henan University of Traditional Chinese Medicine, No. 19 Renmin Road, Zhengzhou, Henan, P.R. China

**Keywords:** diabetic foot ulcers, risk factors, prognostic markers, wound healing

## Abstract

Diabetic foot ulcer (DFU) is a severe and prevalent complication of diabetes mellitus, posing substantial risks to patient health and increasing healthcare burdens globally. These chronic wounds often result from a complex interplay of factors, including neuropathy, ischemia, infection, immune dysregulation, and vascular dysfunction, leading to significant morbidity and, in severe cases, amputation. Effective management of DFUs necessitates a comprehensive understanding of their risk factors and prognostic indicators. This review provides an in-depth examination of the various risk factors and prognostic markers associated with DFUs, integrating insights from cellular mechanisms, emerging biomarkers, omics-based research, serological studies, and clinical assessments. We explore the underlying biological processes, such as the impact of chronic hyperglycemia, oxidative stress, inflammation, impaired angiogenesis, and the role of the microbiome in DFU development. The role of serological markers, including inflammatory and glycemic indicators, in predicting DFU risk and progression is discussed. Additionally, clinical markers and advanced assessment tools, such as ulcer grading systems and imaging technologies, used to evaluate DFU severity and healing are reviewed. By synthesizing these diverse perspectives, this review aims to offer a holistic view of DFU management, highlighting how understanding the interplay of risk factors and prognostic markers can lead to improved prevention strategies and personalized therapeutic interventions.

## 1 Introduction

Diabetic foot ulcers (DFUs) are a severe and prevalent complication of diabetes mellitus, affecting approximately 15–25% of individuals with diabetes at some point in their lives [1]. These chronic wounds are often the result of a complex interplay of factors, including peripheral neuropathy, impaired blood flow, immune dysregulation, repeated trauma, and microbial imbalance, leading to significant patient morbidity and increased healthcare costs. DFUs are associated with a high risk of infection, which can lead to limb amputation if not managed appropriately [2].

The clinical importance of DFUs extends beyond the immediate health consequences, as they contribute to prolonged hospital stays, frequent surgical interventions, and substantial healthcare expenditures [3,4]. Additionally, DFUs can severely impact patients’ quality of life by limiting mobility and increasing the risk of psychological distress. Psychological comorbidities such as depression and anxiety are commonly reported and may negatively influence treatment adherence and wound outcomes. Given these challenges, there is an urgent need for a better understanding of the factors that contribute to the development and progression of DFUs, as well as the indicators that can predict their outcomes [5].

Research into the risk factors and prognostic markers for DFUs is crucial for advancing prevention and treatment strategies. Key risk factors include inadequate glycemic control, which accelerates the formation of advanced glycation end-products (AGEs) and exacerbates oxidative stress; peripheral neuropathy, which impairs sensory perception and increases the risk of unnoticed injuries; and peripheral vascular disease, which compromises blood flow and impedes wound healing [6–8]. Additionally, inflammatory responses play a significant role in the pathogenesis of DFUs, with elevated levels of pro-inflammatory cytokines being associated with poor ulcer outcomes. In addition, impaired angiogenesis and endothelial dysfunction, chronic inflammation mediated by immune cells such as macrophages, and elevated oxidative stress biomarkers (8-hydroxy-2′-deoxyguanosine [8-OHdG], malondialdehyde [MDA]) have been increasingly recognized as central contributors to DFU pathogenesis.

Serological markers such as C-reactive protein (CRP) and glycated hemoglobin (HbA1c) provide valuable information about systemic inflammation and long-term glycemic control, respectively [9–11]. Emerging biomarkers, including microRNAs and cytokine panels, show potential for improving prognostic accuracy. Clinical assessment tools, including ulcer grading systems, pressure mapping, and advanced imaging modalities such as optical coherence tomography and hyperspectral imaging, help evaluate the severity of DFUs and guide treatment decisions. Furthermore, omics-based approaches – especially transcriptomics, proteomics, and single-cell analyses – are offering new insights into cellular heterogeneity and disease mechanisms.

This review aims to provide a comprehensive analysis of the risk factors and prognostic indicators associated with DFUs. By integrating insights into the underlying mechanisms, serological profiles, and clinical evaluation techniques, the review seeks to offer a detailed perspective on how these factors contribute to DFU development and progression. Ultimately, this approach will support the development of targeted interventions and personalized treatment strategies to improve patient outcomes and reduce the burden of DFUs.

## 2 Mechanisms of DFU

DFUs are a multifaceted complication of diabetes mellitus, arising from a combination of metabolic, vascular, immunological, and microbiological disruptions that collectively impair normal cellular and tissue functions. A thorough understanding of these mechanisms is crucial for effective prevention, diagnosis, and personalized treatment [12].

One of the primary contributors to DFU development is chronic hyperglycemia, which significantly impacts cellular function [12,13]. The persistent high blood glucose levels lead to the formation of AGEs. These AGEs result from the non-enzymatic reaction between glucose and proteins, lipids, or nucleic acids [14]. AGE accumulation impairs the extracellular matrix (ECM), activates the receptor for AGEs (RAGE) pathway and triggers inflammation and oxidative stress, all of which exacerbate tissue injury and delay healing [15].

Oxidative stress is another critical factor in the pathogenesis of DFUs. Chronic hyperglycemia increases the production of reactive oxygen species (ROS), which cause cellular damage through the oxidation of lipids, proteins, and DNA. This oxidative damage impairs cellular function and triggers inflammatory responses that further complicate wound healing [16]. Oxidative stress biomarkers such as 8-OHdG and MDA have been identified in DFU tissues and are being explored for clinical relevance.

Inflammation plays a significant role in DFU development and progression. Elevated levels of inflammatory mediators such as tumor necrosis factor-alpha (TNF-α), interleukin-1 beta (IL-1β), and interleukin-6 (IL-6) are commonly observed in DFU wounds [17,18]. These cytokines perpetuate a chronic inflammatory state, which impairs tissue repair by promoting the breakdown of ECM components and hindering normal wound healing processes. Macrophage polarization imbalance (M1 over M2) and impaired neutrophil clearance are now recognized as hallmarks of delayed healing in DFU.

Several interrelated signaling pathways are dysregulated in DFUs, contributing to impaired healing through chronic inflammation, oxidative stress, and tissue remodeling defects. The nuclear factor kappa-light-chain-enhancer of activated B cells (NF-κB) pathway is a central mediator of sustained inflammation in DFU wounds. Hyperglycemia, AGE–RAGE interaction, and ROS can activate the NF-κB complex, which translocates to the nucleus and induces the expression of pro-inflammatory genes, including TNF-α, IL-6, and MMP-9. Persistent NF-κB activation prevents resolution of inflammation and disrupts normal wound healing [19].

The transforming growth factor-beta (TGF-β/Smad) signaling pathway plays a critical role in tissue repair by promoting fibroblast proliferation, ECM production, and re-epithelialization. In DFU, TGF-β signaling is often suppressed or dysregulated, leading to reduced collagen I and III synthesis and impaired granulation tissue formation. Moreover, crosstalk between TGF-β and inflammatory pathways further complicates wound resolution [20].

Vascular impairment is closely associated with defects in the VEGF/PI3K–Akt pathway, which normally promotes endothelial cell survival, proliferation, and angiogenesis. In diabetic conditions, VEGF signaling is attenuated or uncoordinated, impairing neovascularization and oxygen delivery to ischemic tissue [21].

Excessive matrix metalloproteinase-9 (MMP-9) expression, often induced by TNF-α and NF-κB signaling, degrades ECM components such as collagen and laminin, disrupting the structural scaffold required for cell migration and wound closure. The imbalance between MMP-9 and its inhibitor TIMP-1 (tissue inhibitor of metalloproteinases) exacerbates ECM degradation. The Nrf2 pathway, which orchestrates the cellular antioxidant response, is often suppressed in DFU. Nrf2 regulates genes encoding antioxidant enzymes such as HO-1 and NQO1. Its reduced activity in diabetic tissues leads to elevated oxidative stress, contributing to cellular injury and chronic inflammation.

Changes in the ECM also contribute to the pathophysiology of DFUs. Diabetes alters the ECM’s structural and functional properties by disrupting the balance between matrix degradation and synthesis [22]. The impaired ECM remodeling results in a dysfunctional matrix that is less effective in supporting wound healing. Changes in ECM components, such as collagen and glycosaminoglycans, contribute to the chronic nature of DFUs and delay healing.

Neuropathy, a common complication of diabetes, further complicates DFU development. Diabetes adversely affects the production and function of neurotrophic factors essential for nerve growth and repair [23]. Reduced levels of these factors impair nerve regeneration and contribute to peripheral neuropathy, which diminishes sensory feedback. Consequently, patients with diabetic neuropathy are less likely to detect and respond to injuries, increasing the risk of ulceration. Additionally, neuronal apoptosis, or programmed cell death, plays a role in diabetic neuropathy [24,25]. The increased loss of sensory nerves due to apoptosis reduces the ability to sense potential injuries or pressure on the feet. This decreased sensory perception leads to delayed detection of wounds and further promotes the development and persistence of DFUs.

Recent advances in multi-omics technologies, including transcriptomics, proteomics, metabolomics, and single-cell RNA sequencing (RNA-seq), are uncovering new regulatory networks and cell-specific responses. For example, microRNAs such as miR-21 and miR-146a have been shown to modulate inflammation and angiogenesis in DFU contexts. Epigenetic modifications, including DNA methylation and histone acetylation, are also being explored as potential therapeutic targets.

To facilitate clinical translation, it is essential to distinguish biomarkers that are clinically validated from those still in early-stage research. While markers like HbA1c, CRP, and MMP-9 have established roles in assessing DFU severity and prognosis, others, such as exosomal microRNAs or single-cell-derived gene signatures, require further validation.

In summary, the pathophysiology of DFUs involves a complex network of metabolic dysregulation, oxidative stress, chronic inflammation, vascular and neural impairment, immune dysfunction, and microbiome imbalance. Table 1 summarizes the interconnected mechanisms and their contributions to DFU progression.

**Table 1:** Different mechanisms and their impacts on DFUs

Possible mechanism	Description	Impact on DFUs	Reference
Chronic hyperglycemia	Formation of AGEs	Damages ECM, impairs tissue repair, exacerbates inflammation	[26]
Oxidative stress	Increased production of ROS	Cellular damage, triggers inflammation, worsens healing	[27]
Chronic inflammation	Elevated inflammatory mediators (TNF-α, IL-1β, IL-6)	ECM breakdown, impairs tissue repair, hinders healing	[15]
ECM changes	Disruption in ECM degradation and synthesis	Dysfunctional matrix, delays healing	[28]
Neuropathy	Reduced neurotrophic factors, impaired nerve regeneration	Diminished sensory feedback, increased ulcer risk	[14]
Neuronal apoptosis	Increased programmed cell death in sensory nerves	Reduced injury detection, promotes DFU development	[6]

## 3 Serological markers

Serological markers are crucial for assessing disease severity, predicting prognosis, and guiding treatment decisions, helping to optimize patient management and improve clinical outcomes. Serological markers are valuable tools for assessing the risk, prognosis, and management of DFUs. These markers provide insights into the systemic conditions that influence ulcer development and healing [29].

Inflammatory markers are critical in assessing the inflammatory status associated with DFUs, and their role in disease progression is well established. CRP is a widely used inflammatory marker that increases in response to acute and chronic inflammation. In DFU patients, elevated CRP levels often correlate with the severity of inflammation and can indicate poor wound healing outcomes. Monitoring CRP levels helps clinicians gauge the inflammatory response and adjust treatment strategies accordingly [30]. Another important inflammatory marker is the erythrocyte sedimentation rate (ESR), which serves as an indicator of chronic inflammation [31]. Elevated ESR levels reflect ongoing inflammatory processes and provide valuable information regarding the inflammatory burden in DFU patients.

Glycemic markers are integral in understanding the relationship between blood glucose control and DFU risk. HbA1c is a key marker for long-term glycemic control, reflecting average blood glucose levels over the past 2–3 months. High HbA1c levels are associated with an increased risk of DFU development and poor healing outcomes [29,32]. Effective glycemic management plays a critical role in reducing the risk of DFUs and promoting wound healing, as poorly controlled blood glucose impairs immune function and tissue repair mechanisms. Additionally, acute fluctuations in blood glucose levels can significantly exacerbate tissue damage and delay healing, making blood glucose monitoring a critical component of DFU management.

Other serum markers provide further insights into the metabolic and nutritional status of DFU patients. Markers of insulin resistance, such as the Homeostasis Model Assessment of Insulin Resistance (HOMA-IR), are key in evaluating insulin resistance, a common condition in diabetic patients [33,34]. Elevated HOMA-IR levels are linked to an increased risk of DFUs and can help predict ulcer development. Furthermore, vitamin D deficiency has been implicated in both impaired immune function and poor wound healing in DFU patients. Assessing vitamin D levels can provide additional information on the nutritional status and overall health of DFU patients, aiding in the development of more effective treatment plans [35,36].

## 4 Omics markers

Omics technologies offer a comprehensive approach to understanding the molecular and cellular changes involved in DFUs. These technologies analyze large-scale datasets of genes, proteins, and metabolites, providing insights into the complex biological processes underlying DFU pathogenesis [37].

Genomic studies are essential in investigating the role of genetic variations and mutations in DFU risk. By employing genome-wide association studies, researchers have identified several genetic loci associated with diabetes and its complications, including DFUs. These genetic variations can influence susceptibility to DFUs by affecting critical processes such as inflammation, oxidative stress, and ECM remodeling. Identifying these genetic factors not only aids in pinpointing individuals at higher risk but also contributes to the development of more precise, personalized prevention strategies [38].

Transcriptomics, which examines gene expression profiles, provides critical insights into the molecular response to DFUs. Techniques like RNA-seq allow for the simultaneous analysis of thousands of genes. Changes in gene expression related to DFUs often reveal alterations in pathways involved in inflammation, wound healing, and cellular stress. For example, increased expression of inflammatory cytokines and decreased expression of wound-healing genes have been commonly observed in DFU tissues. These data can help identify potential therapeutic targets and biomarkers for managing DFUs [39].

Proteomics provides a detailed analysis of the entire set of proteins expressed in cells, tissues, or organisms, offering deeper insights into the molecular underpinnings of DFU pathogenesis. Mass spectrometry-based proteomics enables the identification and quantification of proteins involved in DFU progression. Proteomic analyses have highlighted several proteins linked to inflammation, oxidative stress, and ECM remodeling. For example, changes in levels of MMPs and TIMPs have been associated with impaired ECM remodeling and delayed wound healing. Proteomics helps uncover biomarkers reflecting disease processes and guides the development of targeted therapies [40].

Metabolomics involves analyzing the complete set of metabolites – small molecules involved in metabolic processes [28]. Profiling metabolites in DFU patients can reveal alterations in metabolic pathways related to glucose metabolism, oxidative stress, and inflammation. Techniques like mass spectrometry and nuclear magnetic resonance spectroscopy are used to identify and quantify these metabolites. Observations of changes in metabolites related to oxidative stress or glycation in DFU patients offer insights into disease mechanisms and help identify novel biomarkers for diagnosis and prognosis.

Lipidomics, the study of lipid profiles, also plays a crucial role in understanding DFUs. Changes in lipid composition and concentrations have been linked to inflammation and oxidative stress in DFUs [41,42]. By analyzing lipid profiles, researchers can uncover specific lipid species altered in DFUs, providing a deeper understanding of lipid-related mechanisms that contribute to disease development and progression.

Overall, omics technologies – encompassing genomics, transcriptomics, proteomics, metabolomics, and lipidomics – offer a powerful toolkit for exploring the molecular and cellular changes associated with DFUs. These approaches provide valuable insights into disease mechanisms, identify potential biomarkers, and hold significant potential for the development of personalized prevention and treatment strategies.

## 5 Clinical markers and assessment indicators

In the management of DFUs, clinical markers and assessment indicators play a crucial role in evaluating the condition and guiding treatment [43]. Foot examination and scoring systems are essential for assessing DFUs. Various ulcer grading systems, such as the Wagner classification and the University of Texas system, provide a structured approach to evaluate the severity of foot ulcers [44]. These systems help standardize the assessment and guide treatment decisions. Additionally, foot pressure assessments measure plantar pressure to identify areas at risk of developing ulcers, aiding in preventive care and reducing ulcer incidence.

**Figure 1:**
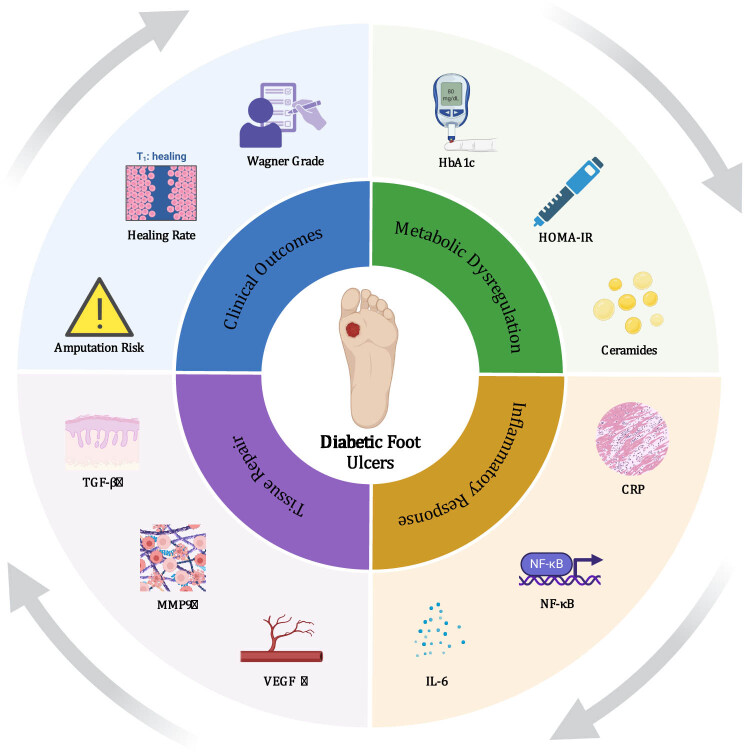
Core pathogenic axis of DFUs.

The status of wound healing is another critical aspect of DFU management. Key indicators include the healing rate, which measures the progress of ulcer closure over time, and the time to healing, which is a crucial clinical parameter reflecting how quickly an ulcer is expected to heal. Monitoring these factors helps in evaluating treatment efficacy and predicting patient outcomes. Table 2 provides a comprehensive summary of serological, omics, and clinical markers used to assess, manage, and understand DFUs Figure 1.

**Table 2:** Overview of markers for DFUs: Serological, omics, and clinical

Category	Marker type	Specific markers	Description	Reference
Serological markers	Inflammatory markers	CRP	Indicates inflammation; elevated levels correlate with DFU severity and poor healing outcomes	[13]
		ESR	Reflects chronic inflammation and overall inflammatory burden	[45]
	Glycemic markers	HbA1c	Reflects long-term blood glucose control; high levels associated with increased DFU risk	[46]
		Blood glucose levels	Acute fluctuations impact DFU risk and healing	[44]
	Insulin resistance	HOMA-IR	Evaluates insulin resistance; elevated levels linked to DFU risk	[29]
	Nutritional mar	Vitamin D	Deficiency associated with poor immune function and wound healing	[31]
Omics markers	Genomics	Genetic variants: TCF7L2, PPARG, KCNJ11	Genes associated with diabetes and DFU susceptibility	[12]
		Inflammatory genes: IL6, TNF, IL1B	Influence inflammation in DFUs	[24]
	Transcriptomics	Inflammatory cytokines: IL6, TNF, IL1B	Increased expression in DFU tissues	[23]
		Wound healing genes: VEGF, MMP-9, TIMP-1	Decreased expression in DFU tissues	[15]
	Proteomics	Inflammatory proteins: TNF-α, IL-1β, IL-6	Associated with inflammation and wound healing	[3]
		ECM remodeling proteins: MMP-9, MMP-2, TIMP-1	Involved in ECM changes	[39]
	Metabolomics	Glucose metabolites: Fructosamine, 3-DG	Indicators of glucose metabolism and oxidative stress	[22]
		Oxidative stress metabolites: 8-OHdG, MDA	Reflect oxidative damage	[25]
	Lipidomics	Inflammatory lipids: Arachidonic acid, 5-HETE	Linked to inflammation and oxidative stress	[25]
		Metabolic lipids: Ceramides, sphingolipids	Associated with metabolic disturbances	[47]
Clinical markers	Foot examination	Wagner classification	System for grading ulcer severity	[46]
		University of Texas classification	Grading system for ulcer severity and depth	[17]
	Foot pressure	Plantar pressure measurements	Identifies areas at risk for ulcer development	[29]
	Wound healing status	Healing rate	Measures ulcer closure progress	[16]
		Time to healing	Reflects how quickly an ulcer is expected to heal	[46]

The interactions between TGF-β, MMP-9, VEGF, IL-6, NF-κB, and CRP play crucial roles in the pathogenesis and healing process of DFUs. TGF-β promotes collagen synthesis during wound healing, while MMP-9 regulates ECM degradation. VEGF enhances angiogenesis to improve local blood flow, IL-6 modulates the inflammatory response, and NF-κB influences immune responses by regulating the expression of inflammatory mediators. CRP, as an acute-phase reactant, reflects systemic changes in the inflammatory state, and these molecules are closely involved in the pathology of DFUs.

## 6 Discussion

DFUs present a complex challenge in diabetes management, with their development and progression influenced by a range of interrelated factors. [45]. The mechanisms underlying DFUs reveal the profound impact of chronic hyperglycemia, which leads to the formation of AGEs. These AGEs accumulate and cause damage to the ECM, disrupting tissue repair and exacerbating inflammation. Concurrently, oxidative stress, characterized by increased production of ROS, further contributes to cellular damage and impairs the wound healing process [48,49]. This combination of AGEs and oxidative stress creates a detrimental environment for wound healing, highlighting the critical need for effective glycemic control and potential antioxidant therapies to manage DFUs [47].

For the mechanism of DFU, multiple interrelated factors contribute to its onset and delayed healing, including metabolic dysfunction, neuropathy, vascular impairment, chronic inflammation, and dysregulated molecular signaling. Persistent hyperglycemia leads to the formation of AGEs, oxidative stress, and impaired cellular repair, while diabetic neuropathy causes sensory loss, motor dysfunction, and autonomic disturbances, increasing the risk of unnoticed injuries and skin breakdown. Concurrently, both macrovascular and microvascular complications result in tissue ischemia and hinder the delivery of oxygen, nutrients, and immune cells. Chronic inflammation, marked by elevated IL-6, TNF-α, CRP, and sustained NF-κB activation, impairs normal wound resolution. Key molecular players such as TGF-β, MMP-9, VEGF, and CRP are involved in ECM remodeling, angiogenesis, and immune regulation but are often dysregulated in the diabetic environment. The wound microenvironment – characterized by hypoxia, high glucose levels, and bacterial biofilms – further disrupts the healing cascade, prolonging the inflammatory phase and impairing tissue regeneration. These complex mechanisms collectively underlie the pathogenesis and therapeutic challenges of DFUs [50].

Peripheral neuropathy complicates DFU management by reducing sensory feedback, making it difficult for patients to detect and respond to injuries. Diabetes-induced damage to neurotrophic factors and increased neuronal apoptosis result in diminished sensory perception. This diminished sensory feedback increases the risk of ulceration and highlights the need for interventions aimed at nerve protection and regeneration. Addressing neuropathy can aid in early injury detection and potentially prevent the development of DFUs [26,51].

Infection and trauma play central roles in the pathogenesis and progression of DFUs [52]. Peripheral neuropathy, a common complication of diabetes, reduces protective sensation and leads to repetitive microtrauma from ill-fitting footwear or unnoticed injuries. Structural deformities and limited joint mobility further exacerbate pressure points on the plantar surface, fostering ulcer formation. Once the protective skin barrier is breached, bacterial colonization can quickly escalate to infection. Infections not only impede wound healing through local inflammation and biofilm formation but also contribute to systemic complications. Severe infections can lead to cellulitis, abscess formation, and osteomyelitis, significantly increasing the risk of lower limb amputation. A prompt, accurate assessment of both trauma and infection is therefore essential to prevent adverse outcomes. Preventive strategies, including patient education, routine foot care, and appropriate footwear, are integral to reducing the incidence and severity of DFU.

Serological markers provide valuable insights into the systemic conditions affecting DFUs. Elevated CRP and ESR are indicators of systemic inflammation and correlate with DFU severity and healing outcomes [27]. Monitoring these markers is essential for assessing the inflammatory burden and adjusting treatment plans accordingly. Glycemic markers, such as HbA1c, reflect long-term blood glucose control and are crucial in managing DFU risk. High HbA1c levels are directly linked to an increased risk of DFUs and poor healing outcomes, reinforcing the need for consistent glycemic management. Additionally, acute fluctuations in blood glucose can exacerbate ulcer development and impair healing, further underlining the importance of stable glucose control in DFU prevention and management.

A comprehensive evaluation of DFUs is crucial for effective management and prognosis. Several clinical scoring systems have been developed to standardize assessment [53]. The Wagner classification remains widely used, focusing primarily on ulcer depth and the presence of gangrene or osteomyelitis. Complementing this, the University of Texas wound classification system incorporates parameters such as infection and ischemia, offering a more detailed stratification. The SINBAD system (Site, Ischemia, Neuropathy, Bacterial infection, Area, and Depth) provides a simplified yet informative approach suitable for resource-limited settings. In addition to these clinical tools, recent advancements in diagnostic imaging, such as thermography, hyperspectral imaging, and MRI, enhance the detection of tissue damage and infection. Furthermore, research into biochemical markers and molecular signatures holds promise for early diagnosis and monitoring. Together, these tools support clinicians in making informed decisions and tailoring treatment strategies.

Omics technologies have revolutionized our understanding of DFUs by providing comprehensive insights into the molecular and cellular changes associated with the condition [54]. Genomic studies have identified genetic variations that influence susceptibility to DFUs, while transcriptomics reveals changes in gene expression related to inflammation and wound healing. Proteomics and metabolomics offer deeper insights into the protein and metabolic profiles associated with DFUs, uncovering biomarkers that reflect disease processes and guide targeted therapies. These technologies enable a more detailed understanding of DFU mechanisms and support the development of personalized treatment strategies that are tailored to the individual patient [55].

Effective management of DFUs involves a multifaceted strategy that targets both the underlying etiologies and the ulcer itself [52]. The initial step typically includes meticulous wound debridement to eliminate necrotic tissue, which is essential for preparing the wound bed and preventing infection. Infection control is also critical and may require systemic or topical antibiotics, depending on the severity and microbial profile. Pressure offloading, particularly using total contact casting, helps redistribute weight and minimize mechanical stress on the affected area. In parallel, maintaining strict glycemic control is fundamental to enhancing the body’s healing capacity. Beyond these standard measures, adjunctive therapies are being increasingly adopted to enhance outcomes. For instance, recombinant growth factors such as platelet-derived growth factor have been employed to stimulate tissue regeneration. Stem cell therapies and bioengineered skin substitutes offer additional regenerative potential, particularly in chronic or non-healing ulcers. Moreover, hyperbaric oxygen therapy has demonstrated effectiveness in improving tissue oxygenation, especially in ischemic or hypoxic wounds. Looking ahead, innovative treatments such as gene therapy and nanotechnology-based drug delivery systems are under active investigation. These emerging approaches hold promise for more targeted and efficient management of DFU. Ultimately, the most effective treatment requires an individualized, evidence-based plan tailored to the patient’s specific risk factors and wound characteristics.

In summary, the interplay of hyperglycemia, inflammation, oxidative stress, neuropathy, and serological markers shapes the development and progression of DFUs. A multifaceted approach integrating insights from mechanistic studies, serological profiles, and omics technologies is essential for advancing both prevention and treatment strategies. Understanding these factors in detail can lead to improved prognostic accuracy and more effective management, ultimately enhancing patient outcomes and reducing the burden of DFUs.

## References

[1] Wang J, Yang X, Zhou T, Ma H, Yuan X, Yan S, et al. Microenvironment of diabetic foot ulcers: Implications for healing and therapeutic strategies. J Res Med Sci. 2025;30:19. 10.4103/jrms.jrms_573_24 PMC12039865 40302998

[2] Lim JZ, Ng NS, Thomas C. Prevention and treatment of diabetic foot ulcers. J R Soc Med. 2017;110(3):104–9. 10.1177/0141076816688346 PMC5349377 28116957

[3] Brocco E, Ninkovic S, Marin M, Whisstock C, Bruseghin M, Boschetti G, et al. Diabetic foot management: Multidisciplinary approach for advanced lesion rescue. J Cardiovasc Surg (Torino). 2018;59(5):670–84. 10.23736/S0021-9509.18.10606-9 29808982

[4] Mishra SC, Chhatbar KC, Kashikar A, Mehndiratta A. Diabetic foot. BMJ. 2017;359:j5064. 10.1136/bmj.j5064 PMC5688746 29146579

[5] Navarro-Flores E, Cauli O. Quality of life in individuals with diabetic foot syndrome. Endocr Metab Immune Disord Drug Targets. 2020;20(9):1365–72. 10.2174/1871530320666200128154036 32003676

[6] Velissaris D, Pantzaris ND, Platanaki C, Antonopoulou N, Gogos C. Procalcitonin as a diagnostic and prognostic marker in diabetic foot infection. A current literature review. Rom J Intern Med. 2018;56(1):3–8. 10.1515/rjim-2017-0039 29028632

[7] Du H, Li S, Lu J, Tang L, Jiang X, He X, et al. Single-cell RNA-seq and bulk-seq identify RAB17 as a potential regulator of angiogenesis by human dermal microvascular endothelial cells in diabetic foot ulcers. Burn Trauma. 2023;11:tkad020. 10.1093/burnst/tkad020 PMC10440157 37605780

[8] Wang Y, Shao T, Wang J, Huang X, Deng X, Cao Y, et al. An update on potential biomarkers for diagnosing diabetic foot ulcer at early stage. Biomed Pharmacother. 2021;133:110991. 10.1016/j.biopha.2020.110991 33227713

[9] Sen P, Demirdal T, Emir B. Meta-analysis of risk factors for amputation in diabetic foot infections. Diabetes Metab Res Rev. 2019;35(7):e3165. 10.1002/dmrr.3165 30953392

[10] Akyuz S, Bahcecioglu Mutlu AB, Guven HE, Basak AM, Yilmaz KB. Elevated HbA1c level associated with disease severity and surgical extension in diabetic foot patients. Ulus Travma Acil Cerrahi Derg. 2023;29(9):1013–8. 10.14744/tjtes.2023.08939 PMC10560815 37681727

[11] Perez-Panero AJ, Ruiz-Munoz M, Cuesta-Vargas AI, Gonzalez-Sanchez M. Prevention, assessment, diagnosis and management of diabetic foot based on clinical practice guidelines: A systematic review. Medicine (Baltimore). 2019;98(35):e16877. 10.1097/MD.0000000000016877 PMC6736276 31464916

[12] Huang F, Lu X, Yang Y, Yang Y, Li Y, Kuai L, et al. Microenvironment-based diabetic foot ulcer nanomedicine. Adv Sci (Weinh). 2023;10(2):e2203308. 10.1002/advs.202203308 PMC9839871 36424137

[13] Mezger PR, Vrijhoef MM, Greener EH. The corrosion behaviour of high-palladium porcelain-bonding alloys. J Dent. 1989;17(1):33–7. 10.1016/0300-5712(89)90005-5 2645331

[14] Guo P, Tu Y, Liu R, Gao Z, Du M, Fu Y, et al. Performance of risk prediction models for diabetic foot ulcer: a meta-analysis. PeerJ. 2024;12:e17770. 10.7717/peerj.17770 PMC11260075 39035162

[15] Dorr S, Lucke-Paulig L, Vollmer C, Lobmann R. Malignant transformation in diabetic foot ulcers-case reports and review of the literature. Geriatrics (Basel). 2019;4(4):62. 10.3390/geriatrics4040062 PMC6961039 31703431

[16] Qi X, Cai E, Xiang Y, Zhang C, Ge X, Wang J, et al. An immunomodulatory hydrogel by hyperthermia-assisted self-cascade glucose depletion and ROS scavenging for diabetic foot ulcer wound therapeutics. Adv Mater. 2023;35(48):e2306632. 10.1002/adma.202306632 37803944

[17] Srivastava P, Sondak T, Sivashanmugam K, Kim KS. A review of immunomodulatory reprogramming by probiotics in combating chronic and acute diabetic foot ulcers (DFUs). Pharmaceutics. 2022;14(11):2436. 10.3390/pharmaceutics14112436 PMC9699442 36365254

[18] Li XY, Zhang XT, Jiao YC, Chi H, Xiong TT, Zhang WJ, et al. In vivo evaluation and mechanism prediction of anti-diabetic foot ulcer based on component analysis of Ruyi Jinhuang powder. World J Diabetes. 2022;13(8):622–42. 10.4239/wjd.v13.i8.622 PMC9412855 36159224

[19] Wang H, Wu S, Bai X, Pan D, Ning Y, Wang C, et al. Mesenchymal stem cell-derived exosomes hold promise in the treatment of diabetic foot ulcers. Int J Nanomed. 2025;20:5837–57. 10.2147/IJN.S516533 PMC12065540 40351704

[20] Wei WH, Bai YL, Zhu D, Zhang JY, Yin QC, Li Q, et al. Dl-3-n-butylphthalide ameliorates diabetic foot ulcer by inhibiting apoptosis and promoting angiogenesis. World J Diabetes. 2025;16(4):101916. 10.4239/wjd.v16.i4.101916 PMC11947905 40236845

[21] Tang G, Wang Y, Deng P, Wu J, Lu Z, Zhu R, et al. Mechanism of dracorhodin in accelerating diabetic foot ulcer healing via the Nrf2 pathway, a network pharmacology, molecular docking and experimental validation. Sci Rep. 2025;15(1):12492. 10.1038/s41598-025-97831-5 PMC11992152 40216975

[22] Hosty L, Heatherington T, Quondamatteo F, Browne S. Extracellular matrix-inspired biomaterials for wound healing. Mol Biol Rep. 2024;51(1):830. 10.1007/s11033-024-09750-9 PMC11263448 39037470

[23] Basra R, Papanas N, Farrow F, Karalliedde J, Vas P. Diabetic foot ulcers and cardiac autonomic neuropathy. Clin Ther. 2022;44(2):323–30. 10.1016/j.clinthera.2021.12.002 34974945

[24] Yao Y, Shi J, Zhang C, Gao W, Huang N, Liu Y, et al. Pyruvate dehydrogenase kinase 1 protects against neuronal injury and memory loss in mouse models of diabetes. Cell Death Dis. 2023;14(11):722. 10.1038/s41419-023-06249-2 PMC10630521 37935660

[25] Huang W, Wu D, Cai C, Yao H, Tian Z, Yang Y, et al. Inhibition of MST1 ameliorates neuronal apoptosis via GSK3beta/beta-TrCP/NRF2 pathway in spinal cord injury accompanied by diabetes. Redox Biol. 2024;71:103104. 10.1016/j.redox.2024.103104 PMC10914584 38430683

[26] Zhu J, Hu Z, Luo Y, Liu Y, Luo W, Du X, et al. Diabetic peripheral neuropathy: Pathogenetic mechanisms and treatment. Front Endocrinol (Lausanne). 2023;14:1265372. 10.3389/fendo.2023.1265372 PMC10803883 38264279

[27] Ibrahim I, Nuermaimaiti Y, Maimaituxun G, Luo X, Maimaituxun M, Akbar A, et al. Neutrophil extracellular traps (NETs) are associated with type 2 diabetes and diabetic foot ulcer related amputation: A Prospective Cohort Study. Diabetes Ther. 2024;15(6):1333–48. 10.1007/s13300-024-01579-6 PMC11096146 38619692

[28] Klashami ZN, Ahrabi NZ, Ahrabi YS, Hasanzad M, Asadi M, Amoli MM. The vitamin D receptor gene variants, ApaI, TaqI, BsmI, and FokI in diabetic foot ulcer and their association with oxidative stress. Mol Biol Rep. 2022;49(9):8627–39. 10.1007/s11033-022-07698-2 35857173

[29] Hoffmanova I, Sanchez D, Szczepankova A, Habova V, Tlaskalova-Hogenova H. Serological markers of intestinal barrier impairment do not correlate with duration of diabetes and glycated hemoglobin in adult patients with type 1 and type 2 diabetes mellitus. Physiol Res. 2022;71(3):357–68. 10.33549/physiolres.934874 PMC9470092 35616045

[30] Altannavch T, Roubalova K, Broz J, Hruba D, Andel M. Serological markers of Chlamydia pneumoniae, cytomegalovirus and Helicobacter pylori infection in diabetic and non-diabetic patients with unstable angina pectoris. Cent Eur J Public Health. 2003;11(2):102–6. 12884557

[31] Mac Mullan PA, Peace AJ, Madigan AM, Tedesco AF, Kenny D, McCarthy GM. Platelet hyper-reactivity in active inflammatory arthritis is unique to the adenosine diphosphate pathway: A novel finding and potential therapeutic target. Rheumatology (Oxford). 2010;49(2):240–5. 10.1093/rheumatology/kep377 19965976

[32] Vujasinovic M, Tepes B, Makuc J, Rudolf S, Zaletel J, Vidmar T, et al. Pancreatic exocrine insufficiency, diabetes mellitus and serum nutritional markers after acute pancreatitis. World J Gastroenterol. 2014;20(48):18432–8. 10.3748/wjg.v20.i48.18432 PMC4277983 25561813

[33] Yu S, Han Z, Li C, Lu X, Li Y, Yuan X, et al. Cross talk between macrophages and podocytes in diabetic nephropathy: Potential mechanisms and novel therapeutics. Mediators Inflamm. 2025;2025:8140479. 10.1155/mi/8140479 PMC12064321 40352596

[34] Yu S, Li Y, Lu X, Han Z, Li C, Yuan X, et al. The regulatory role of miRNA and lncRNA on autophagy in diabetic nephropathy. Cell Signal. 2024;118:111144. 10.1016/j.cellsig.2024.111144 38493883

[35] Sacerdote A, Dave P, Lokshin V, Bahtiyar G. Type 2 diabetes mellitus, insulin resistance, and vitamin D. Curr Diab Rep. 2019;19(10):101. 10.1007/s11892-019-1201-y 31506836

[36] He M, Hou G, Liu M, Peng Z, Guo H, Wang Y, et al. Lipidomic studies revealing serological markers associated with the occurrence of retinopathy in type 2 diabetes. J Transl Med. 2024;22(1):448. 10.1186/s12967-024-05274-9 PMC11089707 38741137

[37] Wigger L, Barovic M, Brunner AD, Marzetta F, Schoniger E, Mehl F, et al. Multi-omics profiling of living human pancreatic islet donors reveals heterogeneous beta cell trajectories towards type 2 diabetes. Nat Metab. 2021;3(7):1017–31. 10.1038/s42255-021-00420-9 34183850

[38] An Y, Xu BT, Wan SR, Ma XM, Long Y, Xu Y, et al. The role of oxidative stress in diabetes mellitus-induced vascular endothelial dysfunction. Cardiovasc Diabetol. 2023;22(1):237. 10.1186/s12933-023-01965-7 PMC10475205 37660030

[39] Zhang K, Peng P, Huang J, Chen M, Liu F, Zhu C, et al. Integrating plasma metabolomics and gut microbiome to reveal the mechanisms of Huangqi Guizhi Wuwu Decoction intervene diabetic peripheral neuropathy. J Ethnopharmacol. 2024;319(Pt 3):117301. 10.1016/j.jep.2023.117301 37820997

[40] Pichu S, Patel BM, Apparsundaram S, Goyal RK. Role of biomarkers in predicting diabetes complications with special reference to diabetic foot ulcers. Biomark Med. 2017;11(4):377–88. 10.2217/bmm-2016-0205 28326825

[41] Walczak-Skierska J, Monedeiro F, Maslak E, Zloch M. Lipidomics characterization of the microbiome in people with diabetic foot infection using MALDI-TOF MS. Anal Chem. 2023;95(44):16251–62. 10.1021/acs.analchem.3c03071 PMC10633811 37877781

[42] Chen H, Liao C, Yang X, Zhou H, Wu Y, Sun Q, et al. Multi-omics analysis revealed the role of CYP1A2 in the induction of mechanical allodynia in type 1 diabetes. Front Genet. 2023;14:1151340. 10.3389/fgene.2023.1151340 PMC10076588 37035728

[43] Maddaloni E, Bolli GB, Frier BM, Little RR, Leslie RD, Pozzilli P, et al. C-peptide determination in the diagnosis of type of diabetes and its management: A clinical perspective. Diabetes Obes Metab. 2022;24(10):1912–26. 10.1111/dom.14785 PMC9543865 35676794

[44] Wagner J, Spille JH, Wiltfang J, Naujokat H. Systematic review on diabetes mellitus and dental implants: An update. Int J Implant Dent. 2022;8(1):1. 10.1186/s40729-021-00399-8 PMC8724342 34978649

[45] Biondo M, Tomasello L, Giordano C, Arnaldi G, Pizzolanti G. The promising approach of 3D bioprinting for diabetic foot ulcer treatment: A concise review of recent developments. Heliyon. 2024;10(17):e36707. 10.1016/j.heliyon.2024.e36707 PMC11395744 39281506

[46] Armstrong DG, Tan TW, Boulton AJM, Bus SA. Diabetic foot ulcers: A review. JAMA. 2023;330(1):62–75. 10.1001/jama.2023.10578 PMC10723802 37395769

[47] Deng H, Li B, Shen Q, Zhang C, Kuang L, Chen R, et al. Mechanisms of diabetic foot ulceration: A review. J Diabetes. 2023;15(4):299–312. 10.1111/1753-0407.13372 PMC10101842 36891783

[48] Wang H, Li N, Ye Y, Zhao N, Liu M, Xu M, et al. Development and validation of the healthcare-seeking intention questionnaire in patients with diabetic high-risk foot. Patient Prefer Adherence. 2024;18:1873–83. 10.2147/PPA.S479644 PMC11404499 39286515

[49] Ringblom A, Ivory J, Adlerberth I, Wold AE, McIntosh C, Wolf A. Wound cleansing solutions versus normal saline in the treatment of diabetic foot ulcers - A systematic review. J Tissue Viability. 2024;33(4):591–7. 10.1016/j.jtv.2024.08.010 39278793

[50] Lv D, Cao X, Zhong L, Dong Y, Xu Z, Rong Y, et al. Targeting phenylpyruvate restrains excessive NLRP3 inflammasome activation and pathological inflammation in diabetic wound healing. Cell Rep Med. 2023;4(8):101129. 10.1016/j.xcrm.2023.101129 PMC10439185 37480849

[51] Sloan G, Selvarajah D, Tesfaye S. Pathogenesis, diagnosis and clinical management of diabetic sensorimotor peripheral neuropathy. Nat Rev Endocrinol. 2021;17(7):400–20. 10.1038/s41574-021-00496-z 34050323

[52] McDermott K, Fang M, Boulton AJM, Selvin E, Hicks CW. Etiology, epidemiology, and disparities in the burden of diabetic foot ulcers. Diabetes Care. 2023;46(1):209–21. 10.2337/dci22-0043 PMC9797649 36548709

[53] Rai V, Moellmer R, Agrawal DK. Clinically relevant experimental rodent models of diabetic foot ulcer. Mol Cell Biochem. 2022;477(4):1239–47. 10.1007/s11010-022-04372-w 35089527

[54] Tanwar AS, Shruptha P, Paul B, Murali TS, Brand A, Satyamoorthy K. How can omics inform diabetic foot ulcer clinical management? A whole genome comparison of four clinical strains of Staphylococcus aureus. Omics. 2023;27(2):51–61. 10.1089/omi.2022.0184 36753700

[55] Wang X, Yuan CX, Xu B, Yu Z. Diabetic foot ulcers: Classification, risk factors and management. World J Diabetes. 2022;13(12):1049–65. 10.4239/wjd.v13.i12.1049 PMC9791567 36578871

